# Impact of Artificial Intelligence on the Knowledge, Attitude, and Performance of Ophthalmology Residents: A Systematic Review

**DOI:** 10.18502/jovr.v20.17029

**Published:** 2025-07-30

**Authors:** Alireza Najafi, Samane Babaei, Mohammad Mehdi Sadoughi, Masomeh Kalantarion, Ali Sadatmoosavi

**Affiliations:** ^1^Department of Medical Education, School of Medical Education and Learning Technologies, Shahid Beheshti University of Medical Sciences, Tehran, Iran; ^2^Ophthalmic Research Center, Research Institute for Ophthalmology and Vision Science, Shahid Beheshti University of Medical Sciences, Tehran, Iran; ^3^Department of Medical Library and Information Sciences, Faculty of Management and Medical Information Sciences, Kerman University of Medical Sciences, Kerman, Iran

**Keywords:** Artificial Intelligence, Attitude, Knowledge, Medical Education, Ophthalmology Residents, Performance

## Abstract

This systematic review investigated the role of artificial intelligence (AI) in the knowledge, attitude, and performance of ophthalmology residents. We conducted a comprehensive systematic search in international databases including PubMed, Web of Science, Scopus, CINAHL (Cumulative Index to Nursing and Allied Health Literature), Education Resources Information Center (ERIC) using keywords “artificial intelligence”, “deep learning”, “ophthalmology”, “ocular surgery”, and “education” and their synonyms. The keywords were extracted from medical research studies published from January 1, 2018 to April 15, 2024. The quality of these studies was evaluated by using the STORBE, JADA, and JBI appraisal tools. Six studies were selected based on the defined criteria. Specifically, five of these studies investigated the effectiveness of AI interventions on the performance of ophthalmology residents in diagnosing myopia, corneal diseases (using a confocal microscope), staging of diabetic retinopathy, abnormal findings in posterior segment ultrasonography, including retinal detachment, posterior vitreous detachment, and vitreous hemorrhage, and 13 fundus diseases. One study investigated the residents' attitudes about the application of an AI model for providing feedback in cataract surgery. All six studies showed positive results. Due to the small number of studies found through our systematic search and the variations in the investigated outcomes and study settings, it was not possible to conduct a meta-analysis. Despite the positive reports on improving the diagnostic performance of residents and their attitude toward the usability of AI models in cataract surgery, it is recommended that more studies be conducted in this area. These studies should replicate previous investigations using similar study settings while maintaining high quality standards and addressing existing limitations.

##  INTRODUCTION

The educational program in ophthalmology residency must transform residents into competent ophthalmologists with the appropriate expertise to independently perform their professional duties. The curriculum should evolve to meet the dynamic needs of society and keep pace with advancements in the field.^[1]^

Ophthalmology embraces new technological advancements and integrates them into clinical practice and education, thereby enhancing patient care and residency training.^[2]^ In recent years, artificial intelligence (AI) has significantly impacted ophthalmology. The field has progressed from automating manual tasks, such as ophthalmic image processing, to deploying machine learning and deep learning.^[3]^ Adopted by several medical institutions worldwide, AI has recently emerged as an integral part of medical education.^[4,5]^ As medical practice undergoes a paradigm shift with the expansion of technology, there is a need to integrate it into the curriculum in order to facilitate the tracking and evaluation of residents.^[6,7,8,9]^

Many studies have focused on the automated diagnosis and grading of ocular diseases based on fundus photographs or optical coherence tomography (OCT) images for conditions such as retinal disorders and glaucoma. These images are also used to predict systemic conditions, such as cardiovascular risks and anemia.^[10,11]^

Several recent studies have explored deep learning-based analysis of cataract surgery videos, which are fundamental to any ophthalmology residency program. Automated processing of surgical videos may enable streamlined postoperative assessments, offering the potential for a more efficient and objective method to evaluate and track residents' performance over time and provide real-time feedback during surgery.^[2]^ Beyond surgical training, AI-based learning has been incorporated into programs that assist residents in reaching clinical diagnoses, yielding better results than traditional education methods.^[12]^

Current studies primarily focus on analyzing the use of AI in medical education. However, very few studies have attempted to categorize AI tools based on their applicability to relevant groups like ophthalmology residents.^[13]^ Additionally, raising awareness about the effectiveness of AI tools for learners' knowledge, attitudes, and performance is crucial for implementing these technology-based tools in medical education.^[14,15]^

Given the importance of applying these developing technologies in ophthalmology education to meet societal needs, and considering the non-convergent and sometimes contradictory results of studies on the effectiveness of AI on ophthalmology residents' knowledge, attitudes, and performance, this study aimed to conduct a systematic review to answer the following question: How effective are AI-based interventions in improving the knowledge, attitude, and the performance of ophthalmology residents?

This review considered ophthalmology residents as the target group and addressed the limitations of previous studies.

##  METHODS

Ethical approval was obtained from the Research Ethics Committee of Shahid Beheshti University of Medical Science (Approval ID: IR.SBMU.REC.1403.001).

### Protocol and Registration

**Figure 1:**
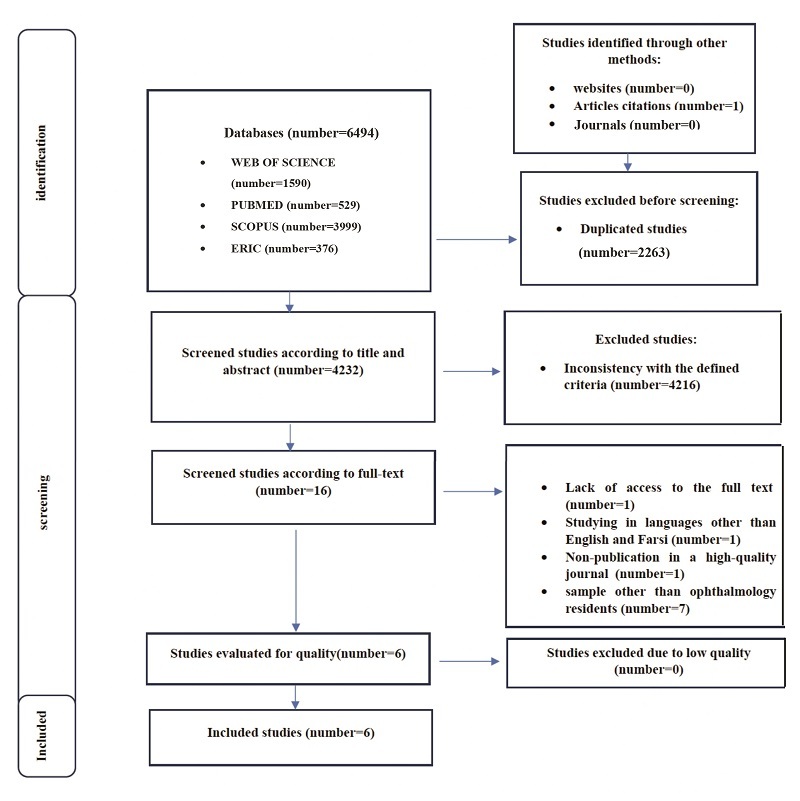
PRISMA diagram of the study.

As demonstrated in Figure 1, this systematic review was conducted according to the PRISMA (Preferred Reporting Items for Systematic Reviews and Meta-Analyses) statement.^[16]^

### Systematic Search Strategy

A comprehensive systematic search was conducted across international literature databases, including PubMed, Web of Science, Scopus, CINAHL (Cumulative Index to Nursing and Allied Health Literature), and Education Resources Information Center (ERIC). Keywords from medical subject headings used in our search included but were not limited to “artificial intelligence”, “deep learning”, “ophthalmology”, “ocular surgery”, and “education”, covering the period from January 1, 2018 to April 15, 2024 (AI had not entered the field of ophthalmology education before 2018.^[17,18]^ For instance, the search strategy for the PubMed database was as follows: (“AI ” OR “CNN ” OR “Artificial Intelligence” OR “Computational Intelligence” OR “Machine Intelligence” OR “Computer Reasoning” OR “Computer Vision Systems” OR “Robotics” OR “expert system” OR “machine learning” OR “neural network” OR “Algorithms” OR “Deep learning” OR “Decision support”) AND (“Ophthalmology” OR “Ocular Surgery” OR “eye surgery” OR “Ophthalmologist” OR “Oculist” OR “eye specialist”) AND (“Education” OR “Instruction” OR “Teaching” OR “Training” OR “Tutoring” OR “Learning” OR “Literacy Program”). Keywords were combined using "OR" and "AND" Boolean operators. The systematic search was independently performed by two researchers. Gray literature, including expert opinions, conference presentations, theses, research and committee reports, and ongoing research, was excluded from this review. Although articles in the gray literature are distributed electronically, they have not been evaluated by a traditional publisher.^[19]^

### Eligibility Criteria

The following inclusion criteria were defined: original articles, interventional studies, English-language publications, population studies consisting of ophthalmology residents, full text availability, and publication in a valid journal. Review articles were excluded from this systematic review.

### Study Selection

Data management for this systematic review was carried out using EndNote, version 21. Two researchers independently conducted the study selection according to the inclusion and exclusion criteria. Initially, titles, abstracts, and full-text articles were evaluated electronically, and duplicates were removed. This process was also performed manually to ensure no data was lost. A third researcher resolved any discrepancy between the two researchers. Finally, references were manually checked to prevent data loss.

### Data Collection Process and Data Items

The following data were extracted from the articles included in this systematic review: the name of the first author, year of publication, location, sample size, sampling method, sample characteristics, study design, duration of participation, independent and dependent variables, type of AI intervention, data collection and measurement tools, aim of the study, main results, and limitations. The data extraction process is shown in Supplementary Table 1.

### Quality Evaluation of Articles and Risk of Bias in Individual Studies

The quality of studies included in this systematic review was evaluated using the STROBE tool (including 22 items) for cross-sectional studies, the JBI tool (including 10 items) for randomized controlled trials (RCTs), and the JADAD tool (including eight items) for studies with pre- and posttest designs. Each item was scored in one of three ways: positive (1 point), negative (no point), and unclear (no point), and finally, the quality was determined based on Table 1.^[20,21,22]^ Also, the articles were examined to identify any conflict of interest and the selection of participants. Two researchers independently extracted the information and evaluated the quality of these studies.

**Table 1:** Score range of appraisal tools.

**Appraisal tool**	**STORBE (%)**	**JBI (%)**	**JADAD**
Low quality	50 ** > **	50 ** > **	4 points ** > **
Average quality	50–70	50–70	–
High quality	70 ** < **	70 ** < **	4 points ** < **

##  RESULTS 

### Study Selection

Initially, after searching the defined databases, a total of 6494 articles were obtained, of which 529 were found in PubMed, 1590 in Web of Science, 3999 in Scopus, and 376 in ERIC. One study was identified through a reference review. Ultimately, after eliminating duplicates, the number of records was reduced to 4232. The screening based on the inclusion and exclusion criteria led to the removal of 4216 articles due to their noncompliance with the defined research objectives. The remaining 16 articles underwent full-text evaluation to check their eligibility for inclusion in our study. Of these, 10 articles were excluded from the study: seven articles involved populations other than ophthalmology residents, one lacked access to the full text in English, one was a thesis and had not been published in scholarly journals, and one was an abstract without access to the full text of the article. Eventually, six articles were included in the study.^[23,24,25,26,27,28]^

### Quality Evaluation and Risk of Bias Within Studies

A single cross-sectional study,^[28]^ assessed based on the STROBE checklist, showed low quality with an average score of < 50% (41% average). Of the two single-group pretest–posttest studies assessed based on the JBI checklist, one^[26]^ featured high quality with an average score of > 70% (77.7% average), and the other^[21]^ had high quality with an average score of 88.8%. ${}^{\text{[57,58]}}$ All three RCTs^[24,25,27]^ were found to be of low quality with an average score of < 4 (average: 3).

### Publication Year and Location of Studies

The six studies included in the present research were conducted in China (*n* = 5)^[21,22,23,24,25]^ and the US (*n* = 1)^[28]^ in 2021 (*n* = 1),^[26]^ 2022 (*n* = 3),^[23,27,28]^ 2023 (*n* = 1),^[24]^ and 2024 (*n* = 1).^[25]^

### Setting of Studies

All eligible studies were conducted in tertiary hospitals, including Renmin Hospital of Wuhan University,^[24,26]^ Peking Union Medical College Hospital,^[23]^ and Hospital of Zhejiang University,^[27]^ as well as the University of Illinois Hospital and Health Sciences Center at Chicago.^[28]^ While five studies were conducted at a single center, one was multicentered and performed across five tertiary hospitals.^[25]^

### Sampling Method of Studies

The sampling methods employed in these studies were generally unclear, with most failing to specify their approach.^[24,25,26,27,28]^ Only one study explicitly mentioned the use of random sampling,^[23]^ highlighting a lack of methodological transparency across the majority of research. This ambiguity raises concerns about the representativeness of the samples and the generalizability of the findings.

### Characteristics of Study Participants

Out of the total 141 participants in the studies, 90.0% (*n* = 127) were ophthalmology residents, 4.9% (*n* = 7) were medical students, 2.3% (*n* = 3) were fellowship students, and 3.1% (*n *= 4) were ophthalmologists. Among 127 ophthalmology residents, 83 individuals (65.3%) from four studies were first and second-year residents,^[23,25,27]^ 30 residents (23.6%) from one study were third-year residents,^[27]^ and the status of 14 residents (11.0%), from two studies, was unclear.^[26,28]^

### Design of Studies

Of the six studies, three were RCTs,^[24,25,27]^ two had a pretest–posttest design,^[23,26]^ and one was a cross-sectional study.^[28]^

### Outcomes Examined in the Studies

#### Effectiveness of artificial intelligence on the performance of ophthalmology residents

Five studies investigated the role of AI in improving the diagnostic performance of ophthalmology residents. More precisely, one study addressed the diagnosis of myopia;^[27]^ one evaluated the diagnosis of corneal diseases using a confocal microscope;^[24]^ one study assessed the diagnosis of diabetic retinopathy grade;^[23]^ one study evaluated the diagnosis of abnormal findings in ocular ultrasound videos (retinal detachment, posterior vitreous detachment, and vitreous hemorrhage);^[26]^ and one study addressed the diagnosis of 13 fundus diseases.^[25]^ Out of a total of five articles focusing on the impact of AI on the diagnostic performance of ophthalmology residents, all five articles ${}^{\text{[23,52--54,56]}}$ confirmed the positive effects of AI on improving the diagnostic performance of ophthalmology residents, and none of the studies reported neutral or negative results in this regard.

#### Effectiveness of artificial intelligence on the attitude of ophthalmology residents

One of the studies investigated the role of AI on the attitude of ophthalmology residents. This study was a follow-up evaluation with a questionnaire, including questions scored on a 4-point scale. The authors evaluated participants' attitudes toward the application of AI-based intraoperative guidance tools for cataract surgery.^[28]^ In total, the authors observed the positive attitude of residents toward the use of AI-based systems, and there was no study with neutral or negative results.

#### Effectiveness of artificial intelligence on the knowledge of ophthalmology residents

Our systematic search found no study concerning the effectiveness of AI on the knowledge of ophthalmology residents.

### Type of Artificial Intelligence Intervention 

Of the five studies focused on improving diagnostic performance, four utilized deep learning-based AI interventions,^[23,24,25,26]^ while one study^[27]^ did not specify the type of AI system implemented. Additionally, one case study^[28]^ explored the effect of AI on the attitude of ophthalmology residents and examined their attitude toward a system based on deep learning.

Furthermore, of the five studies on improving the diagnostic performance of ophthalmic residents, one assessed ocular ultrasonographic videos,^[26]^ three evaluated fundus images, ${}^{\text{[23,54,56]}}$ and one examined corneal images obtained from a confocal microscope.^[24]^ In addition, the study addressing the effect of AI on the attitude of ophthalmology residents investigated surgical films of cataract phacoemulsification procedures. ${}^{\text{[55]}}$

##  DISCUSSION

According to the results obtained from the six final studies based on a systematic search, five studies showed that AI models were effective in diagnosing myopia and corneal diseases (using a confocal microscope), grading of diabetic retinopathy, analyzing abnormal findings (retinal detachment, vitreous detachment, and vitreous hemorrhage) in ocular ultrasound videos, and identifying 13 fundus diseases. Additionally, they confirmed that AI models could enhance the diagnostic performance of ophthalmology residents. All five studies were conducted in China: two in 2022, one in 2021, one in 2023, and one in 2024. Of these five studies on AI's role in improving residents' cognitive performance, three were RCTs and two were pretest–posttest studies. All three RCTs exhibited low quality, and the two pretest–posttest studies had high quality. The observed discrepancy arises from differences in methodological benchmarks and contextual feasibility. RCTs were assessed using tools like the Cochrane Risk of Bias (RoB), which penalizes challenges inherent to educational research (e.g., blinding difficulties, small cohorts). In contrast, pretest–posttest studies were evaluated via JBI checklists, prioritizing the pragmatic aspects (e.g., baseline/post-intervention consistency). RCTs often scored lower due to stringent criteria (e.g., allocation concealment) that conflict with real-world educational constraints, while quasi-experimental designs aligned better with JBI's feasibility-focused metrics. Scoring systems also differ—RCTs use domain-based ratings (low/high risk), while JBI employs percentage scales, amplifying perceived gaps. Thus, lower RCT scores reflect methodological rigor, not inferior evidence value, emphasizing the need to contextualize quality assessments within the study design challenges. In these five studies, among independent variables such as age, sex, previous experience with AI models, and academic year, only the academic year was considered. From this perspective, a total of 127 ophthalmology residents participated in the studies, of whom 83 were in the first or second year (65.3%). Also, 30 residents (23.6%) had completed at least three years of education, and 14 (11%) had an uncertain status. Among the AI-based interventions in five studies, four AI models utilized deep learning, while one had an unclear classification. Yet, all five interventions demonstrated a significant positive effect on the diagnostic performance of ophthalmology residents. The data analyzed by AI models included ocular ultrasound films (to detect retinal detachment, vitreous detachment, and vitreous hemorrhage), eye fundus images (to diagnose the grading of diabetic retinopathy, pathological myopia, and 13 fundus diseases), and corneal images using a confocal microscope (to diagnose corneal diseases). While the duration of residents' participation in the research was not mentioned in one study, the other four reported an average of 21.2 days. In terms of limitations in the five studies, two studies pointed to the limited number of images in some groups of eye diseases (such as some rare diseases) for evaluating the diagnostic performance and training the AI system. Another limitation has been the small number of residents. One study pointed to the variety in residents' areas of expertise, the presentation of nonquantitative primary results in qualitative format (normal versus pathological), and insufficient attention to patients' clinical history and their clinical conditions in diagnostic assessments. Also, four studies did not clarify the process of selecting residents, and one study mentioned the use of random sampling for this purpose. Additionally, the authors in one of these studies did not address conflicts of interest.

AI technology has been integrated into many fields of medicine for diverse applications, including cataract screening and intraocular lens calculations. This acceptance is supported by numerous studies that have examined and reached comparable outcomes regarding its accuracy, sensitivity, and specificity.^[3]^

Numerous meta-analyses confirm the unique potential of AI in predicting and identifying diabetic retinopathy, retinitis pigmentosa, myopia, keratoconus, and age-related macular degeneration, as well as in performing various diagnostic and therapeutic functions.^[29,30,31,32,33]^

However, the results of this systematic search showed that few studies have explored the role of AI in improving diagnostic performance among ophthalmology residents, and a minimal number of studies, such as RCTs, have been reported with sufficient quality based on quality appraisal tools. However, all five studies reviewed here have addressed the effectiveness of AI on the diagnostic performance of ophthalmology residents. They have observed positive outcomes regarding the diagnostic performance of residents in identifying corneal diseases, pathological myopia, diabetic retinopathy, fundus diseases, retinal detachment, posterior vitreous detachment, and vitreous hemorrhage.^[23,24,25,26][27]^

According to the results we obtained from the six final studies, one study investigated the attitude of residents toward the use of AI models in cataract surgery in 2022 in the United States. This survey showed that residents were willing to use the AI model during routine and complex cataract procedures, and more than half of the participants found this tool very useful. In this study, none of the independent variables, such as age, gender, previous history of familiarity with AI model, and academic year, was separately investigated, nor was the type of AI intervention mentioned. Also, the data used by the AI model in cataract surgery were surgical videos of phacoemulsification. The study had a cross-sectional design and indicated a low quality based on our assessment. The limitations in this study raised concerns about the validity of the tool used (questionnaire including questions with a 4-point scale) to measure the attitude of residents toward the application of the AI model due to the lack of a standardized and formulated framework. The other limitation was related to the data type (videos of phacoemulsification cataract surgery) used to train the AI system. Also, this study did not address the method of selecting residents and the conflict of interests.

In the study conducted by Valikodath et al, the attitudes of pediatric ophthalmologists regarding the perceived benefits and concerns of AI in ophthalmology were assessed using a 15-item web-based questionnaire. The findings indicated a generally positive evaluation of these attitudes.^[34]^ Also, Scheetz et al conducted an online survey among fellowship students and residents of three specialized colleges (ophthalmology, radiology/radio-oncology, undifferentiated dermatology) and performed subgroup analysis regarding their attitude toward AI. The majority of participants believed AI would improve their medical field, and the needs of the medical workforce would be affected by this technology in the next decade.^[35]^

However, according to the results of our systematic search, only one study investigated the effectiveness of AI on the attitude of ophthalmology residents. This study showed that the residents were willing to use the defined AI-based system during routine and complex cataract surgery.^[28]^

Our systematic review did not find any study exploring the effectiveness of AI on the knowledge of ophthalmology residents.

Jiang et al investigated the effectiveness of an AI model based on problem-solving learning on the diagnostic and treatment knowledge of congenital cataract among medical students during their ophthalmology internship. In this randomized controlled trial, medical students were divided into two groups: one group was taught using traditional lectures, while the other group received it based on an AI intervention. The results were evaluated according to a pretest–posttest design. After the intervention, a significant difference was observed in the diagnostic knowledge scores, with the group receiving AI-based education scoring higher. However, no significant difference was observed in the knowledge therapy section between the study groups.^[36]^

There were some limitations to conducting this research:

(1) Lack of defined medical subject headings suitable for medical educational dimensions precluded the possibility of a search with a specific scope.

(2) The small number of eligible articles and variations in study settings and final outcomes made it impossible to conduct a meta-analysis.

(3) The citation value of studies for conducting a meta-analysis study was limited due to several factors: insufficient quality in terms of study design, unfavorable results in qualitative evaluation, and lack of transparency of information—such as the procedure for selecting participants—hence increasing the possibility of bias.

(4) The limitation of conducting the systematic search exclusively on studies published in English could also cause information bias.^[37]^

For future research, it is imperative to raise awareness about the effectiveness of AI tools on learners' knowledge, attitude, and performance to facilitate the use of these technologies in medical education.^[14,15]^ While meta-analysis is an efficient approach in systematically obtaining and critically appraising evidence to estimate the benefits or harms of an intervention,^[38]^ it is recommended that future researchers also conduct scoping reviews to summarize and synthesize existing evidence in order to provide insights for practice, programs, and policy development. Additionally, it is suggested that directions for research priorities be outlined to enable meta-analysis studies in the future. This objective could be realized by planning studies centered on previous research in order to maintain consistency in interventions, outcomes, research designs, and study environments.

##  SUMMARY

According to current investigations and available knowledge, no meta-analysis has been undertaken on AI interventions to improve ophthalmology residents' functional, attitudinal, and knowledge outcomes. The phrase “combining apples and oranges” is typically used as a metaphor to describe the problem of combining studies that are different in some ways; however, ignoring important differences among studies can compromise the validity of a meta-analysis.^[39,40]^ Meta-analyses strictly conducted on highly heterogeneous studies may be less interpretable and less useful than initially expected.^[41]^ We found only one study addressing the role of AI in residents' attitudes. Among the five articles concerning AI's role in residents' diagnostic performance, two studies focused on the same eye disease within the same study setting. Considering the limited number of studies exploring the role of AI in the knowledge, attitude, and performance of ophthalmology residents, significant differences in the study settings, and the absence of similar investigated outcomes across at least two studies—the minimum required for a meta-analysis—it was theoretically impossible to conduct a meta-analysis. In fact, at least five studies are needed to perform a meaningful meta-analysis.^[42]^ Based on our extensive search, this is the first systematic review regarding the role of AI in the knowledge, attitude, and performance of ophthalmology residents, and it is hoped that more studies will be conducted in this field in the future.

##  Financial support and Sponsorship

None.

##  Conflicts of interest

None.
